# Successful Surgical Salvage for a Patient with Ruptured Coronary Sinus during Percutaneous Ablation for Atrial Fibrillation: A Case Report

**DOI:** 10.3390/medicina57121302

**Published:** 2021-11-27

**Authors:** Chi-Hao Liao, Chu-Chun Liang, Tzong-Shiun Li, Ying-Chieh Liao, Ying-Cheng Chen

**Affiliations:** 1 Division of Cardiovascular Surgery, Department of Surgery, Changhua Christian Hospital, No. 135 Nanxiao St., Changhua County, Changhua 500, Taiwan; cbm1988x@gmail.com (C.-H.L.); 181028@cch.org.tw (C.-C.L.); 168589@cch.org.tw (T.-S.L.); 2 Division of Cardiology, Department of Internal Medicine, Changhua Christian Hospital, No. 135 Nanxiao St., Changhua County, Changhua 500, Taiwan; 182818@cch.org.tw

**Keywords:** coronary sinus anomaly, coronary sinus rupture, percutaneous ablation, extracorporeal membrane oxygenation

## Abstract

Herein, we describe the rare anatomy of an abnormal shunt from the left atrium to the coronary sinus, which ruptured during a percutaneous ablation for atrial fibrillation. The iatrogenic lesion was successfully repaired after emergent extracorporeal membrane oxygenation set up followed by surgical exploration. The patient’s postoperative course was uneventful, and she was regularly followed up without any complications.

## 1. Introduction

Normally, the coronary venous returns into the coronary sinus (CS) via the four major tributaries, including (1) the great cardiac vein, (2) the anterior interventricular vein, (3) the left marginal and posterior vein, and (4) the middle cardiac or posterior interventricular vein [1]. CS anomalies are rare; however, Mantini et al. discussed and categorized them into four classifications: (1) CS enlargement, (2) CS absence, (3) atresia of the right atrial CS ostium, and (4) CS hypoplasia [2]. We presented a rare anatomy of an abnormal shunt from the left atrium to the CS, which ruptured during a percutaneous ablation for atrial fibrillation (Af). The iatrogenic lesion was successfully repaired after emergent extracorporeal membrane oxygenation (ECMO) set up followed by surgical exploration.

## 2. Case Report

**Figure 1:**
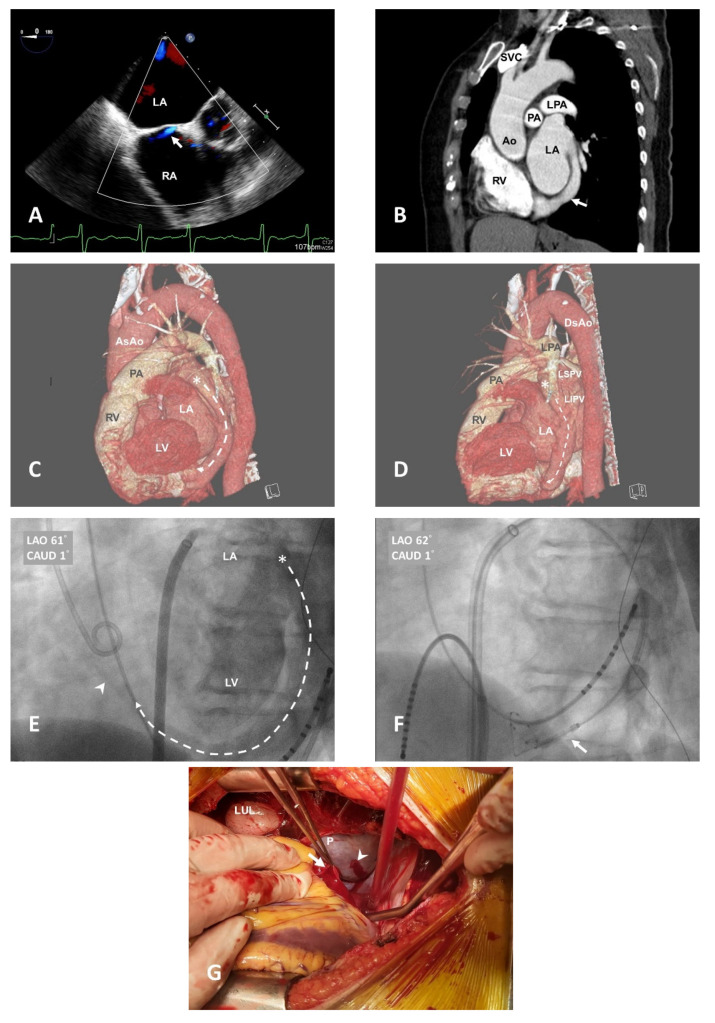
This figure presents the pre-operative images and the intra-operative findings. (**A**) An ASD-like trivial jet (white arrow) but without obvious defect in the transesophageal echocardiography; (**B**) An abnormal shunt originating from the LAPFW into the enlarged CS (white arrow); (**C**) The dashed line indicating the abnormal shunt in the left lateral view of 3D reconstructed CT; (**D**) The left posterior-lateral view of 3D reconstructed CT indicating that the shunt (dashed line) originated from the LAPFW (*); (**E**) Left ventriculography (Supplementary Video S1) by trans-septal needle showed the abnormal shunt (dashed line) inflow (*) and the outflow as CS position (arrowhead); (**F**) The 28 mm cryoballoon (white arrow) was advanced through the shunt for CS ablation (−80 °C, 3 min); (**G**) by elevating the heart, the ruptured CS (white arrow) and previous pericardiocentesis wound (arrowhead) was exposed intraoperatively. LA: left atrium; RA: right atrium; LV: left ventricle; RV: right ventricle; LAPFW: left atrium posterior free wall; Ao: aorta; PA: pulmonary artery; LPA: left pulmonary artery; SVC: superior vena cava; AsAo: ascending aorta; DsAo: descending aorta; LSPV: left superior pulmonary vein; LIPV: left inferior pulmonary vein; LUL: left upper lobe; P: pericardium.

A 58-year-old female patient with hypertension, type 2 diabetes mellitus, and Af history suffered from palpitation and dyspnea upon exertion for months. Pharmacologic treatment had a poor response; thus, she underwent catheter ablation. Before the ablation procedure, the transesophageal echocardiography showed a trivial left-to-right shunt but without obvious atrial septal defect (ASD) (Figure 1A). Additionally, the computed tomography (CT) showed an abnormal vessel originating from the left atrium posterior free wall (LAPFW) (Figure 1B–D), directly shunting to the CS, which was enlarged, and returning to the right atrium (RA). During the ablation procedure using cryoballoon (28 mm Arctic Front Advance™, Medtronic Inc., Minneapolis, MN, USA), strong signals around the bridging vessel were detected (Figure 1E,F) and tried to eliminate them. The cardiologist advanced the cryoballoon into the CS and tried to perform CS ablation (−80 °C, 3 min) but sudden bradycardia and hypotension were noted after balloon deflation and the patient went asystole. Cardiopulmonary cerebral resuscitation (CPCR) was immediately started and venoarterial ECMO support was provided. The transthoracic echocardiography showed a pericardial effusion, under the impression of cardiac tamponade, which caused cardiogenic shock. Thus, pericardiocentesis was performed, which drained out a large amount of fresh blood. Emergent surgical intervention for bleeding control was indicated. After medial sternotomy, cannulation was quickly applied with present femoral cannulas of the ECMO, which was inserted earlier, and her hemodynamic status was stabilized with cardiopulmonary bypass. A clear surgical field was obtained using pump suction and CS rupture was noted, which cause a quick blood loss (Figure 1G). The ruptured vessel wall was successfully repaired using a 5-0 Prolene with pledget. After repairing the lesion, the cardiopulmonary bypass was smoothly weaned off. The ventilator was weaned off on the first postoperative day, and the patient was transferred to the general ward on the third postoperative day. She was regularly followed up without any complications and Af episodes.

## 3. Discussion

The presentation of CS anomaly varies in terms of severity from asymptomatic, to arrhythmia, to heart failure, and depends on the volume and the direction of shunting and electrophysiologic changes [1,2,3]. A CS anomaly with a left-to-right shunt may present an echocardiography result similar to an ASD, just like our patient, which may confuse the surgeon after RA exploration without obvious atrial septum defect but with large CS in the surgical field [2]. Most are asymptomatic and incidentally found by echocardiography, CT, and magnetic resonance imaging for other reasons. Pulmonary isolation alone may be less effective than combined with CS ablation if CS anomaly presents with arrhythmias [4]. In addition to enlarged CS, any other vascular structures may be an important arrhythmogenic origin and concomitant ablation should be demonstrated [5].

This patient visited our cardiologist for persistent Af despite guideline-directed medical therapy and agreed to receive percutaneous ablation therapy. During the ablation procedure, the ruptured CS caused cardiac tamponade and cardiogenic shock. The lesion was successfully repaired and the patient was stabilized using ECMO-assisted CPCR followed by emergent medial sternum exploration. To our knowledge, this is the first case with a CS anomaly that received emergent operation. The cardiologist tried to perform CS ablation through the bridging vein into the CS; however, the CS ruptured after the cryoballoon ablation inside, since the balloon pressure caused a vessel dilatation and rupture. A major complication occurred: however, the patient was successfully resuscitated and the paroxysmal Af was solved after effectively completing the pulmonary vein isolation, abnormal shunt origin isolation, and CS ablation with cryoballoon.

## 4. Conclusions

In summary, CS anomaly may be asymptomatic, which requires no intervention. Pulmonary isolation is considered for patients with Af, as well as CS ablation, whereas shunt ligation is considered to prevent symptom progression for those with heart failure. In addition to pulmonary veins, other cardiac vascular structures, such as enlarged CS, may play an important role in arrhythmogenesis. For these patients, not only should pulmonary vein isolation be performed, but other vascular structure ablations should be demonstrated. During the cryoablation procedure, cryoballoon inflation should be avoided in any vascular structures in order to prevent complications.

## Data Availability

All data are included in the main text.

## Supplementary Materials

The following are available online at https://www.mdpi.com/article/10.3390/medicina57121302/s1, Video S1: Abnormal shunt in left ventriculography.
